# Modified mRNA-Based Therapeutic Strategies for Myocardial Ischemia–Reperfusion Injury

**DOI:** 10.3390/ijms27010055

**Published:** 2025-12-20

**Authors:** Ting Cai, Xiang-Qun Yang

**Affiliations:** 1 School of Gongli Hospital Medical Technology, University of Shanghai for Science and Technology, Shanghai 200093, China; 2 Department of Human Anatomy, Naval Medical University, Shanghai 200433, China

**Keywords:** modRNA, myocardial ischemia–reperfusion injury, gene therapy, cardiovascular diseases, nano delivery

## Abstract

Ischemic heart disease (IHD), the leading causes of cardiovascular morbidity and mortality worldwide, is currently treated though revascularization strategies such as pharmacological thrombolysis, coronary artery bypass grafting (CABG), and percutaneous coronary intervention (PCI). However, the restoration of blood flow often induces cardiac dysfunction, known as myocardial ischemia–reperfusion injury (MIRI). The pathogenesis of MIRI involves a complex, multifactorial process characterized by the interplay of diverse pathophysiological mechanisms, including oxidative stress, intracellular calcium overload, inflammatory cascade activation, apoptosis, autophagy, and microvascular endothelial dysfunction. In recent years, modified RNA (modRNA) technology has emerged as a novel therapeutic strategy for MIRI due to its enhanced molecular stability, reduced immunogenicity, and controllable transient protein expression. Studies have demonstrated that optimized modRNA delivery systems enable efficient, localized expression of therapeutic genes (e.g., antioxidant, anti-apoptotic, and pro-angiogenic factors) at injury sites, significantly mitigating MIRI-associated pathological damage. Nevertheless, significant challenges remain in clinical translation, such as delivery system targeting, transfection efficiency and cytotoxicity. This review focuses on recent advances in the development and application of modRNA-based delivery systems for MIRI treatment. Understanding the molecular mechanisms of MIRI and the structural characteristics and application of modRNA may encourage researchers to explore promising therapeutic modalities for addressing reperfusion-related cardiac injury.

## 1. Introduction

Ischemic heart disease (IHD) remains one of the leading causes of cardiovascular mortality worldwide [1,2]. Pathologically, IHD is characterized by coronary artery occlusion, leading to cellular metabolic dysfunction, ischemic myocardial necrosis, and subsequent cardiac dysfunction of varying severity [3]. In current clinical treatment strategies, revascularization serves as the cornerstone for improving myocardial ischemia, primarily including: pharmacological thrombolysis, percutaneous coronary intervention (PCI), and coronary artery bypass grafting (CABG) [3,4]. These treatment strategies exhibit substantial effectiveness in rescuing at-risk myocardial tissue and enhancing patient prognosis by promptly reestablishing blood perfusion to ischemic heart muscle.

The restoration of blood flow, nevertheless, can induce cardiomyocyte death, clinically recognized as myocardial ischemia–reperfusion injury (MIRI) [5]. The pathogenesis of myocardial ischemia/reperfusion (I/R) injury involves multi-level pathophysiological processes, primarily including the following interrelated mechanisms: excessive production of reactive oxygen/nitrogen species (ROS/RNS); dysregulation of intracellular calcium homeostasis; microvascular and endothelial dysfunction; metabolic disturbances including adenosine triphosphate (ATP) depletion and hydrogen ion accumulation-induced acidosis; activation of innate immune components (neutrophils, platelets, and the complement system); mitochondrial dysfunction; autophagy deregulation; platelet-leukocyte crosstalk [6,7,8,9,10,11]. These pathways collectively form a vicious cycle that perpetuates tissue damage. While ischemic preconditioning represents an effective preventive measure against I/R injury, its clinical application faces multiple limitations [12]. Therefore, developing novel and effective therapeutic approaches is of paramount importance.

**Figure 1:**
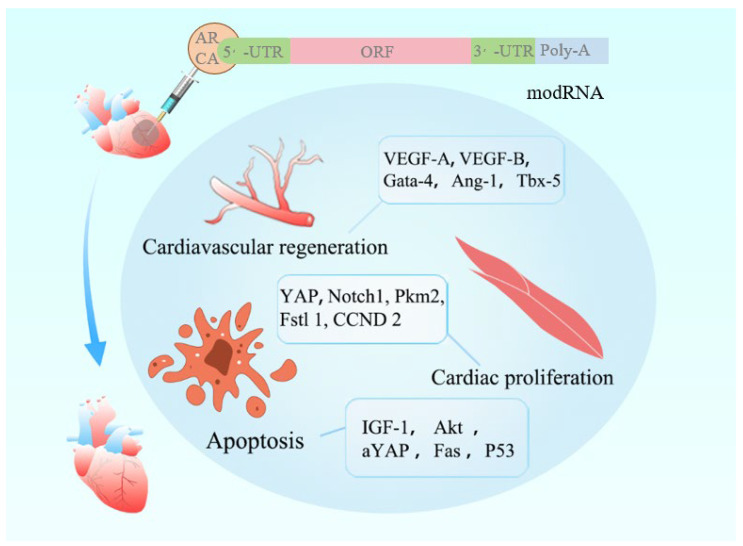
This illustrates a cardiovascular regeneration strategy mediated by modRNA.

The emergence of gene-editing technologies has advanced biomedical research and gene therapy, including mRNA-based, DNA-based, and recombinant protein-based therapies [13]. The traditional exogenous mRNA has high immunogenicity, a relatively short half-life in the body, and the quantity of protein expressed by the mRNA template is limited [14]. This process implies that the unmodified exogenous mRNA has limitations in applications for functional gain studies. Therefore, synthetic modified mRNA (modRNA) as a new type of gene therapy can efficiently, briefly, safely, non-immunogenically and controllably deliver mRNA to the target site, without the risk of genomic integration [15]. With the FDA approval of the mRNA vaccines “Comirnaty” and “Spikevax” for COVID-19, mRNA therapy has become a hot topic in the field of biomedicine [16]. The applications based on mRNA have multiple advantages, including rapid design and production of the required proteins, transient expression, and ease of large-scale production [17]. Therefore, mRNA shows great potential beyond vaccines, such as in the treatment of cardiovascular diseases [18]. Compared with traditional DNA vectors, modRNA as a novel gene therapy tool offers several notable advantages: transient expression characteristics that avoid long-term expression-related side effects; absence of genomic integration risks, ensuring higher safety [18,19]. As illustrated in Figure 1, the therapeutic application of modRNA in cardiovascular diseases is schematically depicted. This review delineates the structural foundations of modRNA, summarizes recent advances in delivery systems tailored for MIRI therapy, and objectively assesses its therapeutic potential alongside prevailing challenges—with a particular focus on delivery efficiency, tissue specificity, and hurdles in clinical translation.

The modRNA molecule features an ARCA cap, 5′-UTR and 3′-UTR elements, an open reading frame (ORF), and a poly-A tail. Structural modifications enhance its stability, fine-tune translational efficiency, and reduce immunogenicity. The encoded therapeutic factors exert regenerative effects on the injured heart through three key mechanisms: ① VEGF-A/B and Gata-4 promote angiogenesis to restore perfusion; ② YAP and Notch1 activate cardiomyocyte cell cycle re-entry, enabling proliferative regeneration; ③ activation of the IGF-1/Akt pathway suppresses apoptosis, thereby limiting myocardial cell death. In animal models, modRNA has demonstrated significant improvement in cardiac function following myocardial infarction and remains under active investigation in animal trials. These findings support its potential as a promising therapeutic modality for cardiovascular regeneration.

## 2. Mechanisms of Myocardial Ischemia–Reperfusion Injury

### 2.1. Oxidative Stress

Oxidative stress represents a fundamental pathophysiological mechanism underlying diverse disease processes, including cancer, cardiovascular diseases, neurodegenerative disorders, and aging [20]. To maintain redox homeostasis, cells employ endogenous antioxidant defense systems that counteract pro-oxidant challenges. Disruption of this delicate equilibrium between oxidant production and antioxidant capacity results in the excessive accumulation of reactive oxygen species (ROS), establishing a state of oxidative stress [21]. ROS are highly reactive free radical molecules, primarily including hydrogen peroxide (H_2_O_2_), superoxide anion (O_2_^−^), hydroxyl radical (OH·), and singlet oxygen (^1^O_2_). Under normal physiological conditions, cellular metabolic processes continuously generate low to moderate levels of ROS, which serve as crucial signaling molecules regulating various cellular biological processes, including cell proliferation, apoptosis, migration, angiogenesis, tumorigenesis, and immune responses [22,23,24]. However, ROS act as a double-edged sword. When excessively accumulated in cells, they trigger oxidative stress responses, causing DNA damage, lipid peroxidation, and protein dysfunction, ultimately leading to pathological changes such as apoptosis or necrosis [24,25,26]. Therefore, maintaining a dynamic balance between ROS production and clearance is critical for preserving cellular homeostasis.

**Figure 2:**
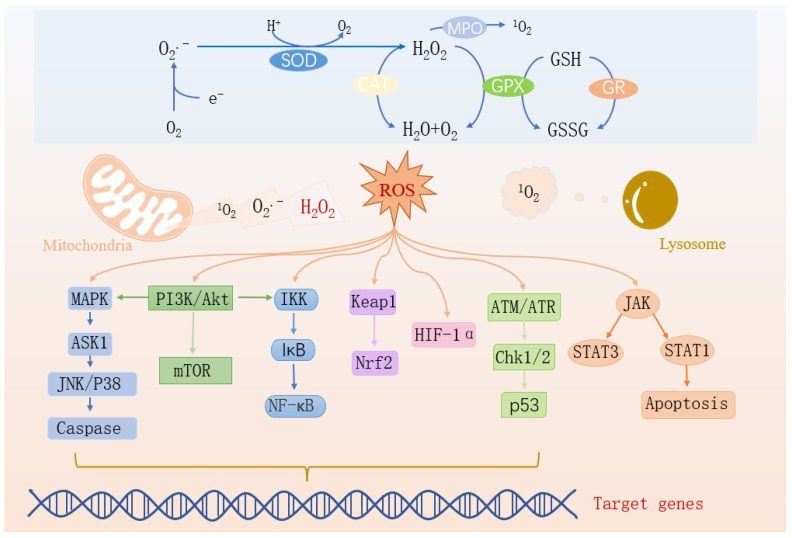
Oxidative stress-related signaling pathways.

Myocardial ischemia resulting from coronary artery occlusion represents a leading cause of cardiovascular mortality, where timely reperfusion remains the primary therapeutic intervention [27]. Paradoxically, during reperfusion, the sudden reintroduction of oxygen into cardiomyocytes leads to an explosive generation of ROS, causing structural and functional damage to ischemic tissues. In addition to producing ROS, cells possess antioxidant systems to eliminate excess ROS [10]. Cellular antioxidants can be divided into two categories: enzymatic antioxidants and small molecules. The enzymatic antioxidant system consists of superoxide dismutase (SOD), glutathione peroxidase (GPx), catalase (CAT), thioredoxin reductase, and glutathione reductase (GR) [28,29]. Non-enzymatic small-molecule antioxidants include vitamins A/C/E, glutathione (GSH), uric acid, thioredoxin, as well as essential trace elements such as manganese, selenium, zinc, and copper, along with ferritin [29]. Current therapeutic strategies targeting myocardial I/R injury focus on antioxidant approaches, including nanoparticles [30], iron-chelating agents [31], traditional Chinese medicine monomers [32], small-molecule ozone [33], and hormones [34]. Figure 2 illustrates the molecular pathways, enzymes, and mechanisms involved in oxidative stress.

When ROS from mitochondria and lysosomes accumulate excessively, they synergistically induce myocardial infarction through multiple pathways: ① Activation of the MAPK→ASK1→JNK/P38→Caspase pathway and the JAK→STAT1 pathway directly induces cardiomyocyte apoptosis. At the same time, ROS damages the mitochondrial membrane, exacerbating cell death. ② Through the PI3K/Akt→IKK→NF-κB pathway, inflammatory factors are upregulated, causing myocardial inflammation, and also disrupting the Keap1-Nrf2 balance, weakening the antioxidant defense. ③ Overactivation of the PI3K/Akt/mTOR pathway induces left ventricular remodeling, interfering with the degradation of HIF-1α, exacerbating hypoxia-reperfusion injury, and inhibiting repair through the ATM/ATR→Chk1/2→p53 pathway, ultimately leading to massive cardiomyocyte necrosis, cardiac dysfunction, and myocardial infarction. They are intertwined to regulate the corresponding genes and apoptosis.

### 2.2. Inflammatory Response

The inflammatory response plays a pivotal role in the pathogenesis of MIRI. Myocardial ischemia and hypoxia can activate immune cells to release pro-inflammatory cytokines, triggering an inflammatory cascade that leads to cardiomyocyte damage and cardiac dysfunction. Conversely, suppression of inflammation has been shown to improve cardiac function [35]. The pathophysiological processes in myocardial tissue involve two distinct types of inflammatory responses: chronic and acute.

Chronic inflammation in MIRI is characterized by sustained activation of inflammatory pathways resulting from immunologic dysfunction in vascular endothelial cells. This persistent activation not only exacerbates endothelial dysfunction but also orchestrates downstream inflammatory cascades, thereby accelerating atherosclerotic progression [36]. In contrast, acute ischemia-hypoxia primarily induces acute inflammation through pro-inflammatory factors released by necrotic cardiomyocytes. During myocardial reperfusion, damaged cells release danger-associated molecular patterns (DAMPs), including immune complexes and inflammatory mediators (e.g., TNF, NLR family proteins, and IL-1β), which activate the innate immune system [37]. These signals are subsequently processed by antigen-presenting cells and transmitted to adaptive immune components (B and T lymphocytes) through pattern recognition receptors (PRRs), initiating targeted clearance of pathogenic molecules [38].

The specific immune response mediated by T and B cells plays a dichotomous role in cardiac ischemia–reperfusion pathology, being implicated in both tissue damage initiation and subsequent reparative processes [39]. CD4^+^ T lymphocytes aggravate cardiac damage following ischemia–reperfusion events through the production of inflammatory mediators, particularly interferon-gamma (IFN-γ) and tumor necrosis factor-alpha (TNF-α) [40]. CD4 + CD25 + regulatory lymphocytes (Tregs) demonstrate substantial protective properties in cardiac tissue. Experimental evidence indicates that depletion of these immunoregulatory cells worsens heart muscle damage and enhances leukocyte migration, while therapeutic administration of Treg populations significantly reduces ischemia–reperfusion-mediated tissue damage [41]. Similarly, B lymphocytes, as crucial mediators of humoral immunity, participate in MIRI pathogenesis through antibody production. Experimental evidence confirms that IgM triggers local myocardial inflammation through complement system activation, thereby participating in the early pathological process of MIRI [42].

Interleukins (ILs), which are important pro-inflammatory cytokines secreted by the innate immune system, including IL-1, IL-2, IL-10, and IL-17, have been demonstrated to participate in host defense responses and infection immunity regulation [43,44,45]. The early stages of cardiac ischemia–reperfusion, cardiomyocytes, endothelial cells, and fibroblasts in myocardial tissue abnormally generate reactive oxygen species (ROS) [46]. These excessive ROS not only directly damage cardiomyocytes and induce apoptosis but also disrupt the phospholipid bilayer structure of cell membranes while stimulating massive TNF-α release from neutrophils [47,48]. Not only exacerbates ischemia–reperfusion injury but also potentiates maladaptive ventricular remodeling, ultimately culminating in progressive cardiac dysfunction. During the initial phase of ischemia–reperfusion injury, ROS and chemotactic cytokines liberated by injured cardiac myocytes initiate complement cascade activation, which orchestrates the chemotactic recruitment of neutrophils to the ischemic myocardium [49]. Mediated by cell adhesion molecules (CAMs) such as ICAM-1 and P-selectin, the infiltrating neutrophils disrupt myocardial microcirculation while releasing substantial quantities of matrix metalloproteinases (MMPs) and reactive oxygen species (ROS), thereby amplifying the inflammatory cascade and establishing the hallmark pathomorphological manifestations of myocardial ischemia–reperfusion injury (MIRI) [49,50].

Mitochondria, the central organelles governing cellular energy metabolism, sustain cardiomyocyte function by generating ATP through oxidative phosphorylation of metabolic substrates. However, during reperfusion, sudden restoration of blood flow induces calcium (Ca^2+^) overload and free radical bursts, which can severely impair mitochondrial function if not promptly cleared [51,52]. Calcium overload activates various proteases and phosphatases that modify key components of the electron transport chain (ETC) and GTPases regulating mitochondrial morphology, promoting abnormal hyperpolarization of mitochondrial membrane potential, explosive ROS generation, and increased outer mitochondrial membrane permeability in ischemic myocardium. These alterations are further amplified when reintroduced oxygen during reperfusion promotes mitochondrial permeability transition pore (mPTP) opening, creating a vicious cycle of mitochondrial dysfunction and cellular injury [53,54].

Mitochondrial quality control (MQC) serves as a critical mechanism for maintaining mitochondrial homeostasis [55]. By regulating mitochondrial structure, function, and metabolism, MQC exerts cardioprotective effects by suppressing cell death pathways and modulating inflammatory responses, thereby alleviating MIRI. Studies suggest that MQC modulation may represent a potential therapeutic target for MIRI [56]. For instance, overexpression of sarco/endoplasmic reticulum Ca^2+^-ATPase (SERCA) can mitigate cardiac microvascular ischemia–reperfusion injury by maintaining MQC [57]. SERCA can improve MQC in I/R injury by inactivating xanthine oxidase, reducing calcium overload, decreasing ROS [1]. These findings collectively suggest that targeted MQC regulation may offer novel therapeutic opportunities for MIRI management.

### 2.3. Autophagy, Apoptosis, and Necrosis

Programmed cellular demise plays a pivotal role in maintaining tissue equilibrium, participating in both cardiac morphogenesis during embryological development and vascular restructuring in mature organisms. Within the cardiovascular compartment, multiple differentiated cell populations demonstrate susceptibility to reperfusion-induced apoptotic signaling cascades [58]. These mechanisms exacerbate cellular injury after reperfusion.

Apoptosis, a predominant form of cardiomyocyte death in reperfusion injury, proceeds through extrinsic or intrinsic pathways. In the intrinsic pathway, a ROS burst during reperfusion leads to cytochrome c release, activating caspase-9/3. The release of cytochrome c is regulated by pro-apoptotic proteins (e.g., Bax, Bak, Bid) and anti-apoptotic proteins (e.g., Bcl-2). After MIRI, the downregulation of Bax inhibitor 1 (BI1) is negatively correlated with endothelial cell apoptosis, microvascular collapse, and mitochondrial damage. BI1 strongly suppresses mitochondrial apoptosis by inhibiting the Syk–Nox2–Drp1 signaling axis, disrupting mitochondrial fission, and maintaining endothelial cell viability. Bcl-2, an anti-apoptotic factor, is expressed in infarcted myocardium but not in non-infarcted tissue. Severe imbalance between pro- and anti-apoptotic proteins on the mitochondrial membrane can lead to mitochondrial depolarization and mitochondrial outer membrane permeabilization (MOMP), ultimately activating the mitochondrial-dependent apoptotic pathway. Additionally, calcium overload and ROS induce mitochondrial swelling, releasing apoptosis-inducing factor (AIF) [59].

The extrinsic pathway also contributes to apoptosis in MIRI. Following MIRI, Fas and FasL expression are markedly upregulated at transcriptional and translational levels. Concurrently, TNF-α pathway activation triggers caspase-8 via reperfusion-induced inflammation, initiating apoptosis [60]. Z-VAD.fmk, a pan-caspase inhibitor, binds to the catalytic site of caspase proteases, reducing caspase-3 levels. Studies in rats with myocardial I/R injury show that Z-VAD.fmk effectively inhibits caspase-3/8 activity, reducing infarct size and improving left ventricular function [61]. Furthermore, miR-484-mediated suppression of caspase-3/9 protects cardiomyocytes from I/R injury during apoptosis [62].

Autophagy is a highly conserved lysosome-dependent degradation process significantly activated under various stress conditions. During myocardial ischemia, energy metabolism impairment and metabolite accumulation inhibit the mTOR signaling pathway, releasing its suppression on the ULK1 complex and thereby initiating autophagy [63,64]. This process selectively eliminates damaged organelles and misfolded proteins, playing a crucial role in maintaining cellular homeostasis. Concurrently, endoplasmic reticulum stress (ERS) pathway activation fine-tunes autophagic activity [65]. Upon reperfusion, autophagy exhibits spatiotemporal heterogeneity: physiological autophagy continues to exert cytoprotective effects by clearing toxic metabolites accumulated during ischemia, whereas excessive autophagy may become detrimental by degrading essential cellular components or activating cell death pathways [63]. This double-edged nature suggests that precise modulation of autophagy based on disease stage may represent a novel therapeutic strategy for ischemia–reperfusion injury.

The AMPK signaling pathway, a central hub in cellular energy homeostasis, plays a pivotal role in autophagy regulation [66]. Numerous studies confirm that AMPK/mTOR pathway activation enhances post-ischemia–reperfusion autophagy, conferring significant cardioprotection [67]. For example, melatonin modulates this pathway to improve mitochondrial dynamics in optic atrophy models, promoting mitochondrial fusion and selective autophagy, thereby attenuating MIRI [68]. Mechanistically, the mitochondrial protein BNIP3 recruits key autophagy effectors (e.g., LC3, Beclin1) to initiate mitophagy [69]. In rat MIRI models, the HIF-1α/BNIP3 pathway is markedly activated, with Western blot and immunofluorescence confirming upregulated HIF-1α and BNIP3 protein levels alongside increased LC3II/LC3I ratios. qPCR further demonstrates coordinated elevation of HIF-1α, BNIP3, and LC3 mRNA [70], indicating that this pathway precisely regulates mitophagy to suppress H9C2 cardiomyocyte apoptosis.

Unlike apoptosis and autophagy, necrosis is an unregulated cell death form triggered by extreme physicochemical stimuli. Notably, inhibition of critical apoptotic or autophagic regulators can divert cellular demise toward necrotic pathways. The RIP kinase family, particularly RIP1/RIP3 complexes, mediates TNF-α-induced necroptosis [71]. Experimental demonstrated that RIP3 exacerbates post-ischemic cardiac remodeling in mice, whereas RIP3 knockout improves cardiac function and reduces ROS-driven inflammation [72]. Key necrotic regulators in cardiac pathology include Tak1, Traf2, CaMKII, microRNAs, HSP90, HAX1, Sirt3, and STAT3. TAK1 suppresses pathological remodeling by modulating the RIP1-caspase8-FADD complex, while RIP3-induced CaMKII phosphorylation increases mitochondrial permeability, promoting necrosis [73]. MicroRNAs like miR-129 exert protection via TLR4/NLRP3 signaling [74]. Mitochondrial proteins also regulate necrosis: HAX-1 inhibits mPTP opening by reducing cyclophilin-D (Cyp-D) levels, and STAT1/3 delays mPTP opening to confer cardioprotection [75]. These findings provide a theoretical basis for necrosis-targeted therapies in MIRI.

## 3. Structural Basis of modRNA

Messenger RNA (mRNA) is a naturally occurring molecule that efficiently and accurately translates genetic information from DNA into proteins to execute physiological functions. In eukaryotic cells, mRNA production involves multiple processing steps, including 5′ capping, splicing to remove non-coding introns, 3′ polyadenylation, and in some cases, RNA editing [76]. 5′ capping occurs when the 5′ end of nascent mRNA (after ~20–30 nucleotides of transcription) is modified with a 7-methylguanosine cap (m^7^GpppN) structure to enhance stability and translational efficiency [77]. Splicing involves the recognition and excision of introns from pre-mRNA by the spliceosome, followed by exon ligation to form mature mRNA. Alternative splicing can generate different transcript variants from a single gene. Finally, polyadenylation adds a 50–250 adenosine tail (poly(A) tail) downstream of the AAUAAA signal sequence to protect mRNA and facilitate translation. However, unmodified mRNA is highly susceptible to degradation by ribonucleases in both extracellular and intracellular environments [78].

modRNA represents a novel class of RNA molecules engineered through chemical modifications of natural RNA. Its in vitro synthesis comprises three key steps: (1) DNA template preparation: Design of DNA templates containing optimized 5′ and 3′ untranslated regions (UTRs) to enhance translation efficiency. The 5′-UTR is a critical regulatory element for protein expression, with sequence features profoundly impacting translational efficacy [79]. For instance, modRNA with GATA2-binding 5′-UTR efficiently directs pluripotent stem cells (PSCs) to differentiate into endothelial cells (ECs) [80]. Tandem repeats in 3′-UTR further boost translation. (2) IVT (in vitro transcription): Synthesis of modRNA from the DNA template. (3) Purification: Isolation of modRNA for downstream applications. modRNA enables rapid protein production in vivo/in vitro while exhibiting gradual degradation and low immunogenicity [18]. Common modifications include: 5-methylcytidine (5meC), N6-methyladenosine (m6A), and N1-methylpseudouridine (m1Ψ). These modifications enhance mRNA functionality [81].

Exonuclease cleavage is the primary pathway of linear mRNA degradation [82]. Therefore, it is critical to protect mRNA transcripts from rapid exonuclease-dependent decay, particularly the deadenylation of the 3′ poly (A) tail, while preserving an efficient cap-dependent translation initiation mechanism [83]. Poly(A)-binding proteins (PABPCs) have a dual role in initiating translation by binding to eukaryotic translation initiation factors (eIFs). It also extends mRNA stability by hiding poly (A) tails to prevent deadenylation [84]. Wang’s team multimerized poly (A) tails by branching topology, with each individual poly (A) tail carrying extensive nuclease resistance modifications that would protect the poly (A) tail integrity from RNA decay and preserve multimerized poly (A)-PABPC1 interactions to prolong translation time [85].

IVT mRNA is designed to mimic mature, processed cytoplasmic mRNA in eukaryotes. Unlike endogenous mRNA (synthesized in the nucleus and exported to the cytoplasm), IVT mRNA must enter the cytoplasm directly from extracellular environments via: (1) endocytosis: membrane encapsulation and internalization into endosomes and (2) endosomal escape: release into the cytoplasm for immediate translation [86]. Further purification to eliminate dsRNA contaminants, thereby minimizing immunostimulation, while maintaining an optimal balance of stability, low immunogenicity, and high translational activity [87]. Collectively, these optimizations enable modified IVT mRNA (modRNA) to effectively correct genetic defects, confer gain-of-function capabilities, and support diverse therapeutic applications in both gene therapy and disease prevention.

## 4. Delivery Vehicles for modRNA

Appropriate delivery carriers are essential for the therapeutic use of mRNA, since they prevent RNase-mediated degradation of mRNA, control innate immune responses against mRNA, and deliver mRNA to the desired site. modRNA delivery systems can be broadly categorized into viral and non-viral vectors. Viral vectors such as adenoviruses, adeno-associated viruses (AAVs), and lentiviruses demonstrate high transduction efficiency but suffer from limitations including restricted insertion capacity, strong immunogenicity, and potential carcinogenic risks [88,89]. In contrast, non-viral vectors like lipid nanoparticles (LNPs) and polymeric nanoparticles offer advantages such as unrestricted gene size capacity, ability to transfect non-dividing cells, and enhanced safety profiles. The characteristics of different delivery modRNA carriers are summarized in Table 1. The unique lipid core structure of LNP systems effectively protects modRNA from nuclease degradation, demonstrating promising therapeutic potential [89,90]. In addition, multiple micelles (CMs) prepared by mixing mRNA with PEG polycation block copolymer with a core–shell structure containing PEG shell and an mRNA-containing core are capable of delivering mRNA by attenuating innate immune responses to mRNA and preventing Toll-like receptor recognition of mRNA [78]. However, studies reveal that nanoparticle-encapsulated modRNA may exhibit lower translational efficiency compared to naked modRNA, which can achieve protein expression within 10 min when delivered in sucrose-citrate buffer [91], highlighting the need to balance protection efficacy with translational efficiency.

To enhance modRNA translation efficiency, researchers have systematically optimized mRNA structural domains. Modifications to the 5′ and 3′ untranslated regions are particularly crucial, as they regulate translation initiation complex assembly and microRNA-mediated mRNA stability to influence protein expression [92]. Studies show that incorporating α- and β-globin mRNA 3′-UTR significantly extends modRNA half-life and improves translation efficiency [93]. Notably, replacing conventional artificial 5′-UTR with the Ces1d gene’s 5′-UTR doubled protein expression levels in myocardial infarction models [77]. These optimized UTR structures not only perform exceptionally in cardiac tissue but also enhance protein translation efficiency in other organs under ischemic conditions.

Regarding delivery methods, intramyocardial and intravenous injections each have distinct characteristics. Previous studies indicate that intramyocardial stem cell injection better facilitates cardiac functional recovery as the delivered cells remain localized [94]. However, this approach remains invasive and may cause epicardial and ventricular wall damage. Compared to intramyocardial injection, intravenous administration can suppress prolonged inflammatory processes and allows for repeated dosing [95]. To address the critical challenge of cell-specific delivery, researchers have developed sophisticated solutions. The Magadum team created a regulatory system based on archaeal L7Ae protein that incorporates cardiomyocyte-specific microRNA recognition elements, enabling targeted gene expression exclusively in cardiomyocytes [96,97]. This precision control technology prevents off-target effects in non-cardiac cells, establishing a new paradigm for cardiac regenerative therapy. Future research should focus on developing safer non-viral vectors; optimizing delivery routes to balance efficiency and safety; refining cell-specific expression systems; and establishing standardized, cost-effective production processes to fully realize modRNA’s tremendous potential in gene therapy.

**Table 1:** Delivery vehicles and their characteristics.

Delivery Vehicle	Composition/Properties	Advantages	Limitations	References
Lipid Nanoparticles (LNPs)	Cationic lipids, phospholipids, cholesterol, PEG-lipids forming uniform lipid core	High encapsulation efficiency; good cellular uptake; scalable production (e.g., COVID-19 mRNA vaccines); clinically validated	Limited cardiomyocyte targeting (primarily accumulates in fibroblasts); may cause mild inflammation	[98,99,100]
Polymeric Nanoparticles	PEI, etc., via electrostatic encapsulation	Tunable structure; biodegradable (good biocompatibility); sustained release	Lower transfection efficiency than LNPs; some polymers (e.g., PEI) show cytotoxicity	[101,102]
Viral Vectors	AAV, lentiviruses carrying modRNA (non-genomic integration)	High transduction efficiency	Immunogenicity risk; limited cargo capacity (<4.7 kb); preexisting antibodies may neutralize vectors (30–50% AAV neutralization rate)	[88,103]
Exosomes	Natural nanovesicles (30–150 nm) with targetable surface peptides	Extremely low immunogenicity; natural membrane penetration; endogenous (avoids phagocytic clearance)	Low yield/purification challenges; inconsistent drug loading	[104,105,106]
Naked modRNA + Buffer	Sucrose-citrate buffer system (unencapsulated)	Fastest translation (protein expression in 10 min); no carrier toxicity; lowest cost	Highly susceptible to nuclease degradation; lacks targeting; only suitable for local injection	[91]

## 5. Applications of modRNA in MIRI Therapy

Following myocardial ischemia–reperfusion injury, cardiomyocyte proliferation is significantly impaired due to persistent inflammatory responses, pathological tissue remodeling, and the development of a fibrotic microenvironment. Stem cells—undifferentiated cells with self-renewal and multilineage differentiation capacities—contribute to cardiac repair primarily through endothelial differentiation, paracrine factor secretion, and immunomodulation [107]. However, their transdifferentiation into functional cardiomyocytes remains controversial. Despite promising preclinical and clinical results, stem cell therapy faces critical limitations including poor cell survival, restricted differentiation potential, safety concerns, and ethical issues before widespread clinical adoption [108].

**Figure 3:**
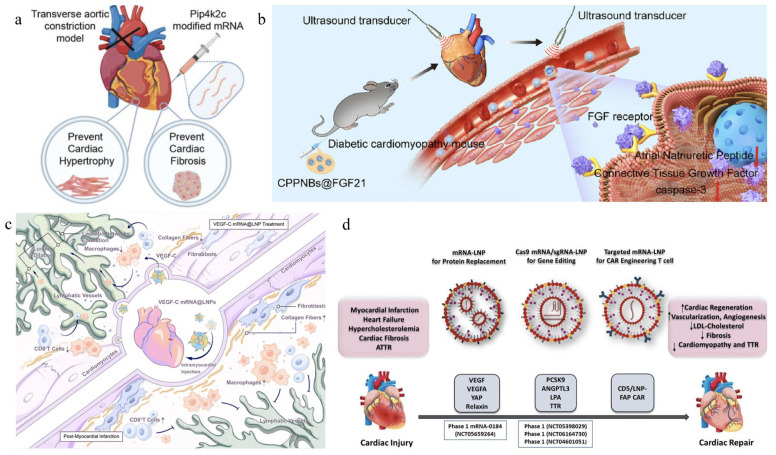
The application of different mRNA in cardiac treatment. (**a**) The Pip4k2c-modified treatment significantly improved cardiac function, reversed cardiac hypertrophy and myocardial fibrosis, and enhanced survival rates in models. (**b**) Poly (lactic-co-glycolic acid) (PLGA) nanobubbles doped with perfluoropropane (C3F8) and polyethyleneimine (PEI) (termed CPPNBs) were synthesized via a double emulsion evaporation method. FGF21 was efficiently loaded into the nanobubbles through electrostatic interactions, resulting in CPPNBs@FGF21. Upon application of low-frequency ultrasound (LFUS), CPPNBs@FGF21 enable targeted delivery of FGF21 to myocardial tissue via ultrasound-triggered cavitation, thereby exerting cardioprotective effects. (**c**) Intramyocardial delivery of VEGF-C mRNA-loaded lipid nanoparticles (LNPs) robustly promotes lymphangiogenesis, attenuates inflammatory cell infiltration, and suppresses pro-inflammatory and fibrosis-associated signaling pathways. (**d**) This study investigates mRNA-LNP-based therapeutics for cardiovascular diseases and further explores the application of LNP-mediated mRNA delivery in CAR T-cell therapy, as well as CRISPR/Cas genome editing for cardiovascular disease interventions [110,111,112].

In contrast, modRNA technology emerges as a compelling alternative, leveraging its high-efficiency protein expression and tunable temporal kinetics to enhance myocardial regeneration. Notably, modRNA-encoded proteins become detectable within 3 h post-intraventricular injection, peak at 18 h, and gradually decline over 6 days—precisely covering critical post-ischemic time windows [109]. This platform shows particular promise in addressing post-reperfusion complications including fibrosis, apoptosis, and necrosis through multi-target interventions, as shown in Figure 3.

### 5.1. Cardiomyocyte Proliferation and Regeneration

Experimental studies demonstrate that intramyocardial delivery of modRNA encoding non-glycosylated follistatin-like 1 (Fstl1) and pyruvate kinase muscle isozyme 2 (Pkm2) promotes cardiac proliferation and regeneration in murine ischemia models [113,114]. These modRNAs incorporate m1Ψ, anti-reverse cap analog (ARCA), and poly-A tail modifications. While glycosylated hFstl1 fails to induce cardiomyocyte proliferation, its non-glycosylated counterpart achieved via N180Q mutation enhances cardiomyocytes proliferation in vitro [106]. Conversely, cardiomyocyte-specific Pkm2 knockout reduces cell numbers, cell cycle progression, and myocardial size. Pkm2 modRNA gain-of-function suppresses oxidative stress-induced damage via β-catenin and anabolic pathways, improving cardiac function and survival [115].

The cardiomyocyte-specific modified mRNA translation (CM SMRT) system further refines targeting specificity [116]. This innovative approach utilizes two modRNA constructs: one encoding Lin28a (a known stimulator of cardiomyocyte proliferation) and another expressing archaeal L7Ae protein regulated by cardiomyocyte-specific miR-1 and miR-208. This dual-component system ensures exclusive protein expression in cardiomyocytes, with Lin28a exerting therapeutic effects through Let-7 microRNA suppression—a negative regulator of cardiomyocyte proliferation acting via c-MYC, HMGA2, and K-RAS pathways.

### 5.2. Cardiovascular Regeneration

Several studies have investigated angiogenic gene therapy approaches, with particular focus on vascular endothelial growth factor A (VEGF-A) as a key therapeutic candidate for promoting neovascularization in ischemic tissues [117]. Intramyocardial administration of modRNA encoding angiogenic factors VEGF/VEGF-A in myocardial infarction murine models significantly enhanced microvascular network formation, as evidenced by increased capillary density and vascular maturation. This therapeutic intervention concurrently reduced fibrotic scar formation, augmented cardiac functional recovery, and improved overall survival rates. VEGF-A modRNA has shown remarkable potential in post-reperfusion vascular regeneration [118]. Subcutaneous injection of Matrigel-embedded VEGF-A modRNA-transfected human Isl1+ progenitors enhances their proliferation/survival and promotes cardiac endothelial differentiation in NOD/SCID mice [119]. The modified VEGF-A mRNA (containing m5C, ΨU, ARCA, poly-A tail, and optimized UTR) significantly improves cardiac function and survival in porcine ischemia models through enhanced vascular endothelial differentiation and reduced fibrosis.

**Figure 4:**
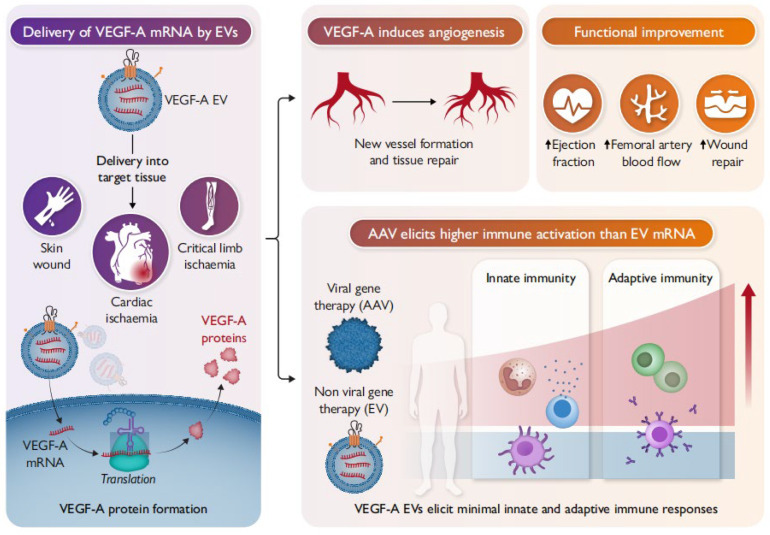
Delivery of VEGF-A mRNA by EVs.

Figure 4 Cellular nanoporation (CNP) enables large-scale loading of full-length VEGF-A mRNAs into secreted EVs. Delivery of VEGF-A mRNA loaded EVs (VEGF-A EVs) to mouse models of ischaemia translated into functional proteins at the target site, resulting in improved functional recovery. Compared to AAV, repeated in vivo administrations of VEGF-A EVs were not observed to cause irritation, allergic reactions, or other immune activation, making them safer and more effective in delivering therapeutic effects [106].

### 5.3. Anti-Apoptotic Strategies

Given the susceptibility of post-injury cardiomyocytes to apoptosis, modRNA-mediated transient expression of protective proteins via IGF-1, TLR, and sphingolipid signaling pathways offers crucial therapeutic benefits [120]. IGF-1 ligand-receptor interaction triggers receptor autophosphorylation, activating PI3K/AKT (through Thr308/Ser473 phosphorylation) and ERK pathways. This cascade upregulates BCL-2, FOXO1/FOXO3a while downregulating BAD, collectively suppressing apoptosis and promoting survival [121]. Cardiac-specific IGF-1 receptor transgenic mouse studies confirm these cardioprotective effects. Intramyocardial IGF-1 modRNA (modified with m5C, ΨU, ARCA, poly-A tail, and optimized UTR) delivery reduces apoptosis through enhanced Akt phosphorylation and suppressed caspase-9 activity.

The Hippo-yes-associated protein (YAP) is a transcriptional coactivator that promotes cell proliferation and survival. YAP is inhibited by kinases such as Mst1/2-Sav kinase complex and Lats1/2 kinase. This pathway is essential for cardiac development [122]. Transient YAP expression via modRNA technology inhibits cardiomyocyte necrosis, neutrophil infiltration, and pathological remodeling in murine I/R models, partially through TLR signaling modulation [123,124]. Mechanistically, YAP suppresses cardiac TLR expression and subsequent inflammatory cytokine production, promoting cardiomyocyte survival.

### 5.4. Anti-Fibrotic Strategies

Myocardial fibrosis is characterized by excessive extracellular matrix deposition, leading to myocardial stiffness and pathological scarring. In vivo engineering of chimeric antigen receptor (CAR)-T cells using mRNA-based platforms offers significant advantages, including streamlined manufacturing processes and transient, controllable transgene expression [125]. Emerging evidence demonstrates that fibroblast activation protein (FAP)-targeted chimeric antigen receptor (CAR) T cells—generated either through in vitro transduction of isolated T cells using lentiviral vectors or via in vivo delivery using T cell-specific lipid nanoparticles (LNPs)—can effectively attenuate cardiac fibrosis and restore cardiac function in mouse models of hypertensive heart injury [126,127].

**Figure 5:**
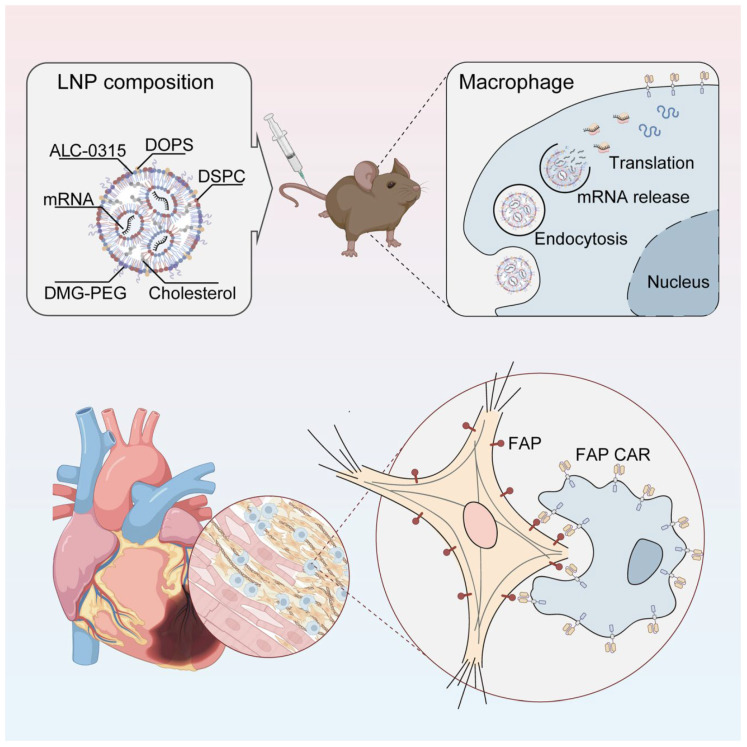
LNP–FAP CAR enables the generation of functional CAR macrophages. These engineered macrophages exhibit specific phagocytic activity against fibroblasts overexpressing fibroblast activation protein (FAP), effectively reducing the population of activated cardiac fibroblasts, attenuating myocardial fibrosis, and significantly improving cardiac function [128].

Lipid nanoparticles (LNPs) encapsulating fibroblast activation protein (FAP)-targeted chimeric antigen receptor (CAR) mRNA (LNP–FAP CAR) were employed to generate FAP CAR macrophages. These engineered macrophages were generated both in vitro and in vivo and demonstrated the ability to specifically recognize and phagocytose FAP-positive activated cardiac fibroblasts. As shown in Figure 5, treatment with LNP–FAP CAR significantly attenuated myocardial fibrosis and improved cardiac function following myocardial ischemia/reperfusion (I/R) injury [128].

## 6. Challenges and Future Perspectives of modRNA in MIRI Therapy

modRNA as a promising therapeutic strategy for MIRI, addressing critical limitations of conventional DNA- and protein-based therapies. Its transient protein expression profile eliminates the need for sustained protein production, offering distinct advantages. However, several critical challenges in delivery efficiency, tissue specificity, and immunogenicity must be resolved to fully realize its clinical potential for cardiovascular applications [129].

For successful clinical application, modRNA must overcome multiple biological barriers: targeted delivery to specific tissues, cellular uptake, endosomal escape, cytoplasmic release, and efficient translation [130]. The therapeutic efficacy is highly dependent on this sequential cascade, with failure at any stage potentially compromising treatment outcomes. Current limitations include nonspecific uptake and premature clearance of systemically administered modRNA, as well as the lack of tissue-specific targeting capabilities compared to viral vectors containing tissue-specific promoters. The transient expression characteristic of modRNA presents a double-edged sword—while reducing long-term risks, the short duration may limit efficacy for certain therapeutic applications. Additional unresolved challenges include determining optimal non-invasive delivery routes, establishing minimum effective doses, and developing cost-effective production methods.

While intracardiac injection currently represents the highest delivery efficiency, its invasive nature necessitates the development of non-invasive, targeted delivery systems. Essential research priorities include optimizing controlled release mechanisms following endocytosis and establishing robust dose–response profiles. Looking ahead, the field is advancing toward precision medicine approaches that harness single-cell sequencing technologies to enable patient-specific modRNA therapeutics. Furthermore, strategic combination with complementary treatment modalities may unlock synergistic benefits, potentially transforming therapeutic outcomes for cardiovascular diseases.

While current applications of modRNA in treating MIRI remain limited, these preliminary findings provide valuable insights and novel conceptual frameworks for future therapeutic development. For instance, Glutathione peroxidase 4 (GPX4) has the function of converting lipid peroxides to lipid alcohols and is a key regulator to inhibit ferroptosis. Previous studies have shown that MI/R-triggered ferroptosis occurs simultaneously with GPX4 inhibition. The decrease in GPX4 levels coincides with the onset of ferroptosis during myocardial ischemia–reperfusion [131]. In contrast, increasing GPX4 levels effectively alleviated myocardial injury and enhanced cardiac function. Interferon gene stimulator (STING) antagonism can promote myocardial ferroptosis. So modified GPX4 mRNA may provide an attractive therapeutic target for the modulation of ferroptosis that occurs during MI/R [132]. Stromal cell-derived factor-1α (SDF-1α), VEGF, etc., are promising targets for therapeutic angiogenesis [133]. Additionally, appropriate vehicles, such as AAV, nanoparticles, and LNPs, are crucial to delivering disease-specific genes to the myocardium. Combining genetic modification and delivery materials could maximize the potential utility of modRNA for gene therapy.

## 7. Conclusions

modRNA represents a transformative therapeutic platform for cardiovascular diseases, offering several advantages over conventional approaches. Its ability to induce transient, titratable protein expression makes it particularly suitable for applications requiring short-term protein production, such as cardiomyocyte proliferation and cardiac reprogramming. The technology’s improved safety profile, scalability, and cost-effective manufacturing potential.

Through precise modulation of key gene expression, modRNA effectively mitigates myocardial damage and facilitates tissue repair. However, there are still challenges in the optimization of delivery systems and the improvement of safety for mRNA-based therapies. Currently, research on mRNA-based treatments for cardiovascular diseases is in the preclinical stage. Future studies on the therapeutic and application aspects of mRNA require the improvement of delivery methods and enhancement of safety. It is necessary to encourage researchers to explore more promising treatment approaches to address heart damage.

## Data Availability

The original contributions presented in this study are included in the article. Further inquiries can be directed to the corresponding author.

## Abbreviations

Ischemic heart disease (IHD), percutaneous coronary intervention (PCI), coronary artery bypass grafting (CABG), myocardial ischemia–reperfusion injury (MIRI), ischemia/reperfusion (I/R), reactive oxygen/nitrogen species (ROS/RNS), adenosine triphosphate (ATP), modified RNA (modRNA), hydrogen peroxide (H_2_O_2_), superoxide anion (O_2_^−^), hydroxyl radical (OH·), and singlet oxygen (^1^O_2_), superoxide dismutase (SOD), glutathione peroxidase (GPx), catalase (CAT), glutathione reductase (GR), danger-associated molecular patterns (DAMPs), pattern recognition receptors (PRRs), interferon-gamma (IFN-γ), tumor necrosis factor-alpha (TNF-α), Interleukins (ILs), cell adhesion molecules (CAMs), matrix metalloproteinases (MMPs), electron transport chain (ETC), mitochondrial permeability transition pore (mPTP), Mitochondrial quality control (MQC), sarco/endoplasmic reticulum Ca^2+^-ATPase (SERCA), Bax inhibitor 1 (BI1), mitochondrial outer membrane permeabilization (MOMP), apoptosis-inducing factor (AIF), endoplasmic reticulum stress (ERS), cyclophilin-D (Cyp-D), endothelial cells (ECs), 5-methylcytidine (5meC), N6-methyladenosine (m6A), N1-methylpseudouridine (m1Ψ), Poly(A)-binding proteins (PABPCs), adeno-associated viruses (AAV), lipid nanoparticles (LNPs), Hippo-yes-associated protein (YAP), Glutathione peroxidase 4 (GPX4).
